# Determinants of the Intention to Use Performance-Enhancing Substances Among Portuguese Gym Users

**DOI:** 10.3389/fpsyg.2019.02881

**Published:** 2020-01-14

**Authors:** Ana Sofia R. Tavares, António Fernando Rosado, João Marôco, Luis Calmeiro, Sidonio Serpa

**Affiliations:** ^1^Faculty of Human Kinetics, University of Lisbon, Lisbon, Portugal; ^2^H&TRC–Health & Technology Research Center, ESTeSL – Lisbon School of Health Technology, Polytechnic Institute of Lisbon, Lisbon, Portugal; ^3^William James Centre for Research, ISPA – University Institute of Psychological, Social and Life Sciences, Lisbon, Portugal; ^4^School of Applied Sciences, Abertay University, Dundee, United Kingdom; ^5^Institute of Environmental Health, Faculty of Medicine, University of Lisbon, Lisbon, Portugal; ^6^Lusophone University, Faculty of Physical Education and Sport, Lisbon, Portugal

**Keywords:** gender, gym users, performance-enhancing substances, psychological strategies, social-cognitive determinants, structural equation modeling

## Abstract

The purpose of this study is to examine the determinants of the intentions to use prohibited performance-enhancing substances (PES) and to test the Theory of Planned Behavior’s usefulness in predicting self-reported PES use in both genders. A convenience sample of Portuguese gym users (*n* = 453) completed an anonymous web-based survey. Structural equation modeling, multigroup analysis, and *t*-test with the Welch correction for heterokedastic variances were used. At the structural level, results support attitudes, beliefs, and subjective norms in predicting intentions to PES use in gym users, with subjective norms being its strongest predictor. Moreover, results showed a significant association between self-reported PES use and intentions to use. The predictive model was invariant across genders; however, compared to males, females believed less in the performance-enhancing effects of PES, were less prone to the influence of significant others, and had weaker intentions to use these substances. Psychological strategies should be based on subjective norms, alongside beliefs and attitudes, toward PES use as these variables influence the intention to use PES in this particular population.

## Introduction

Nowadays, the use of prohibited performance-enhancing substances (PES) to improve one’s ability – commonly known as doping – is widespread across many levels of sport and exercise participation (Goulet et al., 2010; Ntoumanis et al., 2014). At professional and Olympic/Paralympic levels, athletes are responsible for confirming that the substances that they consume are not on the World Anti-Doping Agency (WADA) Prohibited List, which is published yearly. At recreational level, no such regulation exists, although Denmark is one of the few countries with drug control procedures in gyms and fitness centers (Thualagant and Pfister, 2012). According to WADA (2015, p. 18), “doping is defined as the occurrence of one or more of the anti-doping rule violations set forth in article 2.1 through article 2.10 of the Code.” However, this official definition of doping was developed in the context of elite sports to prevent the utilization of methods or substances designed to improve performance; hence, it may not be relevant for gym users. Indeed, the use of illegal substances in fitness contexts generally has esthetic purposes aimed at improving body appearance (Thualagant, 2012). Additionally, the health risks gym users are exposed to are potentially more severe than for athletes because consumption is often done in the absence of regulatory guidelines or medical supervision. The long-term use of these substances (e.g., anabolic-androgenic steroids or AAS, stimulants, erythropoietin, human growth hormone, diuretics) without proper control has been associated with several physical disorders (e.g., metabolic, endocrine, infectious, hepatic, renal, cardiovascular disorders) and psychological symptoms (e.g., depressive symptoms, antisocial and violent behaviors, suicidality) that may lead to irreparable health consequences (Baron et al., 2007; Pope et al., 2014). Therefore, while the term doping is mostly associated with competitive sports, the term performance-enhancing substances will be adopted in this article to refer to their use in the fitness context where there is no competitive performance goal.

In a recent systematic review, it was shown that among gym/fitness center users, the prevalence of reported PES use (namely, AAS) across 26 studies ranged from 4.7 to 70%. Furthermore, the majority of AAS users had limited knowledge of these substances and often underestimated their side effects. The presence of high prevalence rates coupled with a lack of knowledge of the risks associated with the use of PES may represent a public health concern (Tavares et al., 2019a). Therefore, researchers have highlighted the importance of promoting knowledge of psychosocial factors that predict doping intentions and behavior (e.g., Ntoumanis et al., 2014). Such importance rests on the assumption that behavioral choices are affected by personal control factors, social influences, and belief systems concerning the consequences of the behavior (Lucidi et al., 2008).

According to Petroczi and Aidman (2009), doping in sport is a predetermined and intentional behavior predicted by pro-doping attitudes. Therefore, to effectively prevent PES use in gym/fitness contexts, theories of health behavior need to be considered; these theories provide a framework for identifying the determinants of behavior (Lucidi et al., 2004) and are typically rooted on social-cognitive and motivational models from social psychology (Chan et al., 2015). Specifically, integrative models that derive from general models of behavioral prediction, such as the Theory of Planned Behavior (TPB; Lazuras, 2016), Sports Drug Control Model, Life Cycle Model, Trans-contextual Model, or the Theory of Triadic Influence (Lazuras, 2016; Kavussanu and Ring, 2017), have been applied to the study of doping in sport. All of these models emphasize the role of decision making, arguing that doping use is goal-directed, intentional, and self-regulated. However, none of these models incorporate measures of contextual influences (e.g., sociocultural, socioeconomic) or ways of analyzing the effects of these contexts on doping decisions. This is problematic as these contexts have a profound effect on PES initiation (Lazuras, 2016).

The TPB (Ajzen, 1991) is one of the most commonly used frameworks to describe individuals’ doping intentions and behavior (Chan et al., 2015) because it includes a series of non-volitional behaviors (Goulet et al., 2010; Zelli et al., 2010). According to Lazuras et al. (2010), the TPB has been applied to samples of adolescents, non-professional athletes, and gym users to study PES use (e.g., Lucidi et al., 2008; Wiefferink et al., 2008), which illustrates its suitability as a theoretical framework for doping research in populations other than elite athletes.

The TPB, an extension of the theory of reasoned action (TRA; Ajzen and Fishbein, 1980), considers the role of personality factors and social influences in the prediction of behavioral intentions and behavior (Ntoumanis et al., 2013). The theory suggests that the intention to perform a specific behavior is the immediate antecedent of that behavior, which in turn is influenced by three conceptually independent variables (Ajzen, 2002; Chan et al., 2015): attitudes toward the behavior (i.e., the favorable or unfavorable evaluations of performing the behavior); subjective norms (i.e., the perceived social pressure to perform or not to perform the behavior), and perceived behavioral control (PBC; the perceived controllability of the behavior).

The TPB’s constructs (attitudes, subjective norms, and PBC) are supported by corresponding salient beliefs – behavioral, normative, and control beliefs, respectively (Ajzen, 1991). Attitudes toward the behavior are determined by specific behavioral beliefs, which reflect the “perceived likely consequences of engaging in PES use behavior, weighted by an evaluation of these consequences” (Kirby et al., 2016, p. 8). Subjective norms are the result of the interaction between normative beliefs, which assess the individual’s perceptions of the expectations of significant others, and the individual’s motivation to comply with such expectations. Finally, PBC is thought to be a function of control beliefs (i.e., factors that can enable or inhibit the target behavior and consequent behavior perceived impact) weighted by the perceived power one has over of each factor (Ajzen, 1991; Kirby et al., 2016). As attitudes, subjective norms, and PBC “may serve as proxy indices of behavior through their direct relationship with behavioral intention and indirect relationship with behavior,” assessing an individual’s behavioral intention may be sufficient to better understand PES use and its antecedents (Kirby et al., 2016, p. 8).

In addition to the central variables of TPB, researchers have also explored the demographic predictors of PES use, and results have demonstrated significant associations between current use, past use, and gender (Ntoumanis et al., 2014). Although findings concerning gender as a predictor of vulnerability to use PES are equivocal (Devcic et al., 2018), it appears that males tend to be more susceptible to doping or PES use than females, placing them at a greater risk of adverse health events (Pedersen, 2010; Ntoumanis et al., 2013; Backhouse et al., 2015). Previous studies in competitive athletes and gym users showed that current or past behaviors predict both intentions to use PES and future behavior (Armitage and Conner, 2001; Ajzen, 2002; Wiefferink et al., 2008; Lazuras et al., 2010). Indeed, gym users who were already PES users tend to have more favorable attitudes toward their use. In addition, these individuals were at a higher risk of repeated use (Wiefferink et al., 2008; Dunn et al., 2009). Finally, the intentions to use these substances effectively predicted the corresponding behavior (Goulet et al., 2010).

The present study explores the determinants of intentions to use PES in a sample of gym/fitness center users in Portugal. Research on this population is limited, thus this study may provide comprehensive information about intentions toward PES use to inform the development of prevention programs and protect gym/fitness users’ health (Backhouse et al., 2014; Molero et al., 2017). To examine the psychosocial mechanisms that may lead to PES use, we adopted an approach based on TPB. This approach has been the prevailing framework in doping behavior research in sport (e.g., Lucidi et al., 2008; Goulet et al., 2010; Ntoumanis et al., 2013) and gym/fitness contexts (Wiefferink et al., 2008). For this purpose, we used the “Questionnaire of Attitudes toward Doping in Fitness” (QAD-Fit; Tavares et al., 2019b), which is anchored in the TPB. Although the concept of perceived behavioral control distinguishes this theory from the TRA, this contruct was not measured in the present questionnaire for two reasons. First, the factor perceived behavior control was removed from the analysis because it did not meet confirmatory factor analysis (CFA) criteria (Tavares et al., 2019b). These difficulties with the PBC factor are not unique. A perceived behavioral control factor did not emerge in Serpa Leitão et al.’s (2001) study with athletes and non-athletes. In addition, Lucidi et al. (2008) also showed a weak influence of PBC on intentions using a similar scale.

Second, it was expected that the majority of the subjects in the present study had not had previous experience with PES use. According to Ajzen (2001), perceived behavior control only has an influence on intention and behavior if individuals have a reasonable idea of the actual control they have over it.

The aims of the present study were threefold: (1) to evaluate whether the intention to use PES in a sample of gym users could be predicted by the variables considered within the TPB, (2) to examine TPB’s usefulness in predicting PES in both genders, and (3) to evaluate whether intentions to use PES predict PES use. Based on the reviewed literature, it is expected that: (1) the TPB variables will significantly predict PES use intentions in our sample of gym/fitness center users; (2) the predictive model in study will be invariant across genders, and males will have more favorable attitudes, subjective norms, beliefs, and intention to use PES than females; and (3) intentions will predict self-reported PES use.

## Materials and Methods

### Participants

A sample consisting of 453 Portuguese gym/fitness center users (age range = 16–79; mean age = 35.64 years; SD = 13.08) participated in this research. Of all participants, 277 were females (61.1%), 175 were males (38.6%), and one did not respond (0.3%). Participants were involved in several gym activities: 57% cardio fitness, 56.5% recreational bodybuilding, 27.8% stretching, and 27.2% localized gymnastics. At the time of data collection 11.1% (*n* = 50) of the participants reported current use of PES. Although we used a non-probabilistic convenience sampling, the sample size required for this study was calculated by an *a priori* sample size calculator for structural equation models (Soper, 2017). Considering a model with four latent variables and 16 observed variables, to achieve a power of 0.9, an anticipated effect size of 0.2 with a probability level of 0.05, it was determined that 434 participants would be required. The participants met the following inclusion criteria: participation in any gym activity; being over the age of 16; being able to read; and having access to a smartphone, tablet, or PC to enter in the online platform through which the survey was administered. This investigation was not deemed to cause physical or psychological harm to respondents. However, participants were informed that they could find some questions uncomfortable; hence, the informed consent form made clear that participation was voluntary and that their responses were confidential and anonymous. Furthermore, they had the right to leave any questions unanswered or withdraw from the study at any time, without having to provide reasons or suffering any consequences. Participants who were 16–17 years of age were informed that they should discuss the study with their parents or legal guardians and were asked to confirm in the online consent form that they had done so. This procedure was sanctioned by the university ethics committee.

### Measures

#### Self-Reported Use of PES

To assess self-reported PES use, participants answered “yes” or “no” to the following question: “As part of your practice, have you ever taken performance-enhancing substances?” Participants were categorized into two groups: (a) those who have never used PES and (b) those who had used or currently use PES. The reason for this categorization was to determine the risk of PES use among these groups (Barkoukis et al., 2013). As a criterion for the PES, the WADA Prohibited List was followed; therefore, vitamins and dietary supplements were not considered.

#### QAD-Fit

The variables derived from the TPB (attitudes, subjective norms, beliefs, and PES use intention) were measured by the QAD-Fit. This instrument contains 16 items that measure four dimensions: attitudes (assessed by the mean of five items; e.g., “Selling PES should be punished”), subjective norms (assessed by the mean of three items; e.g., “I would take PES, if most people I know approved of it”), beliefs (assessed by the mean of three items; e.g., “Performance-enhancing substances help to improve physical abilities”), and intention (assessed by the mean of five items; e.g., “I would take PES to achieve my goals in the practice of physical activity”). All items were answered on a 7-point Likert-type scale ranging from (1) *totally disagree* to (7) *totally agree*. A higher score indicates a more positive attitude toward PES consumption. QAD-Fit is a psychometric valid instrument for Portuguese gym/fitness users. Specifically, the total composite reliability (CR) was 0.85, with values of 0.74 for beliefs, 0.84 for attitudes, 0.86 for subjective norms, and 0.97 for intentions (Tavares et al., 2019b).

### Procedures

After the approval of the study protocol (no. 38/2017) by the Ethical Committee of the Faculty of Human Kinetics of the University of Lisbon, participants were recruited by gyms and fitness center clubs in the Great Lisbon area, who advertised the study through their institutional e-mail and Facebook accounts. Prior to data collection, the survey web link directed potential participants to an informed consent page, where additional information was provided and the methods to ensure anonymity and confidentiality were described. Then, a web-based survey administered via REDCap software (Version 5.11.4, Vanderbilt University) was utilized to collect (1) demographic data; (2) self-reported use of PES (doping behavior); and (3) attitudes, subjective norms, beliefs, and PES use intention. The questionnaires took approximately 15 min to complete. The data collection procedures did not allow any data to be traced back to individual participants or their Internet providers. Also, encryption procedures were utilized during data transfer.

### Data Analysis

A two-step approach to maximum likelihood (ML) structural modeling was performed using IBM SPSS AMOS 24.0 (Analysis of Moment Structures; IBM Corp., Armonk, NY, United States). First, a confirmatory factor analysis (CFA) was conducted to estimate the measurement components of the constructs in the TPB. Indicators loaded on their underlying factors, and inter-factor correlations were allowed. The factors’ measurement errors were estimated as well. Assessment of model fit was based on multiple indicators (Hu and Bentler, 1999; Marsh, 2007), namely, chi-square (χ^2^) statistical test, the ratio of chi-square to its degree of freedom (χ^2/^df), comparative fit index (CFI), parsimony comparative fit index (PCFI), parsimony goodness-of-fit index (PGFI), Tucker-Lewis index (TLI), standardized root means square residual (SRMR), and root mean square error of approximation (RMSEA). The fit of the model was considered good for CFI above 0.95, TLI greater than 0.95, SRMR less than 0.08, and RMSEA below 0.06 (Bentler and Bonett, 1980; Blunch, 2008; Arbuckle, 2009; Byrne, 2010; Kline, 2011; Marôco, 2014).

The composite reliability (CR) was used as a measure of internal consistency of the factors, where values greater 0.70 indicate good reliability (Fornell and Larcker, 1981). Convergent validity and discriminant validity were assessed to test construct validity. To evaluate convergent validity, the average variance extracted (AVE) was estimated, where values greater than 0.50 show convergent validity (Fornell and Larcker, 1981). The condition to ensure discriminant validity was that the AVE for each construct was larger than the interconstruct squared correlation (Fornell and Larcker, 1981).

Second, the structural model (whereby the measurement and structural components were simultaneously estimated) was performed to test the first and second hypotheses. Each factor was specified to predict its respective indicators. Then, attitudes, subjective norms, and beliefs – allowed to freely intercorrelate with one another – were set to be predictive of intentions to use PES in the global sample (*n* = 453).

Once successfully ascertained that the structural model could predict intentions to use PES, a multigroup analysis was conducted to identify differences on the path coefficients among models for the gender groups. Analysis of the invariance for the structural model was performed with maximum likelihood estimation with the Emulisrel6 method by constraining a series of nested models to test, sequentially, for configural, metric (factor loadings), scalar (items’ intercepts), and structural weights (for the causal model). Invariance was probed using the ΔCFI criterion (Cheung and Rensvold, 2002) over the Δχ^2^ criterion (Satorra and Bentler, 2001) given the fact that the latter is too restrictive (Marôco, 2014).

To compare TPB variables (i.e., attitudes, subjective norms, and beliefs) and intentions to PES use mean scores between genders, a *t*-test with the Welch correction for heterokedastic variances was used on the latent factor scores. To compute the standardized effect size, Cohen’s *d* was calculated (Hong et al., 2003).

Considering that self-reported PES use is a dichotomic variable (i.e., yes/no), to test the hypothesis that the use of PES would be predicted by intentions, a probit regression of PES use on intentions was performed with the Lavaan package (Rosseel, 2012) using the diagonally weighted least squares (DWLS) estimator for categorical variables, from the R statistical software (R Core Team, 2019).

## Results

### Preliminary Analyses

Summary measures, skewness (*sk*), kurtosis (*ku*), and a histogram for each of the 16 items are presented (Table 1) and were used to access distributional properties and psychometric sensitivity. Absolute values of *sk* smaller than 3 and *ku* smaller than 7 were considered indicative of no strong deviations from the normal distribution (Finney and DiStefano, 2013; Marôco, 2014). The Mardia’s coefficient (133.18) exceeded minimum values for the multivariate normality, reason why a Bollen-Stine bootstrap (B-S) on 2000 samples was used for subsequent analysis (Nevitt and Hancock, 2001).

**TABLE 1:** Questionnaire of Attitudes toward Doping in Fitness items descriptive statistics.

**QAD-Fit-16 Items**	**Missing**	**Complete**	***n***	**Mean**	**SD**	**p0**	**p25**	**p50**	**p75**	**p100**	***Sk***	***Ku***	**Histogram**
Qad3	0	453	453	2.18	1.79	1	1	1	3	7	1.35	0.48	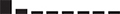
Qad5	0	453	453	2.28	1.93	1	1	1	3	7	1.39	0.61	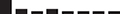
Qad13	0	453	453	4.13	1.93	1	3	4	6	7	–0.08	–0.98	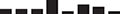
Qad16	0	453	453	2.13	1.73	1	1	1	3	7	1.39	0.65	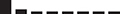
Qad17	0	453	453	4.01	1.69	1	3	4	5	7	–0.34	–0.64	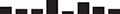
Qad18	0	453	453	2.15	1.69	1	1	1	3	7	1.35	0.63	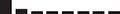
Qad20	0	453	453	2.16	1.73	1	1	1	3	7	1.32	0.42	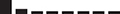
Qad21	0	453	453	3.39	1.86	1	1	4	5	7	0.02	–1.22	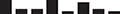
Qad22	0	453	453	1.66	1.14	1	1	1	2	7	1.88	2.94	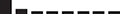
Qad24	0	453	453	2.27	1.81	1	1	1	3	7	1.28	0.34	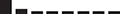
Qad25	0	453	453	3.37	1.96	1	2	3	5	7	0.42	–0.94	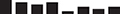
Qad28	0	453	453	2.9	1.64	1	1	3	4	7	0.24	–1.12	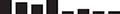
Qad30	0	453	453	2.81	1.86	1	1	2	4	7	0.86	–0.33	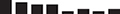
Qad31	0	453	453	1.77	1.38	1	1	1	2	7	1.93	3.03	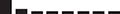
Qad32	0	453	453	2.39	1.81	1	1	2	3	7	1.26	0.51	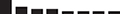
Qad34	0	453	453	1.55	1.15	1	1	1	2	7	2.22	4.31	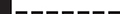

*SD, Standard Deviation; *SK*, skewness; *Ku*, kurtosis; p, percentile.*

### General Results

Table 2 shows the descriptive statistics of the whole sample for attitudes, subjective norms, beliefs, and intention. Mean values are below 4 for all variables, showing that gym users were against the use of PES.

**TABLE 2:** Correlation, means, standard deviations, CR, AVE, and discriminant validity.

**Factors**	**1**	**2**	**3**	**4**
(1) Intentions	1			
(2) Attitudes	0.21^∗∗^	1		
(3) Subjective norms	0.50^∗∗^	0.42^∗∗^	1	
(4) Beliefs	0.35^∗∗^	0.43^∗∗^	0.53^∗∗^	1
Mean	2.18	2.99	1.66	3.43
Standard deviation	1.66	1.47	1.08	1.40
CR	0.97	0.83	0.85	0.73
AVE	0.87	0.50	0.70	0.49
MSC	0.25	0.19	0.28	0.28

*CR, composite reliability; AVE, average variance extracted; MSC, maximum squared correlation. ^∗∗^Bolen-Stine *p* < 0.001 at two-tailed.*

### The Measurement Model

**FIGURE 1:**
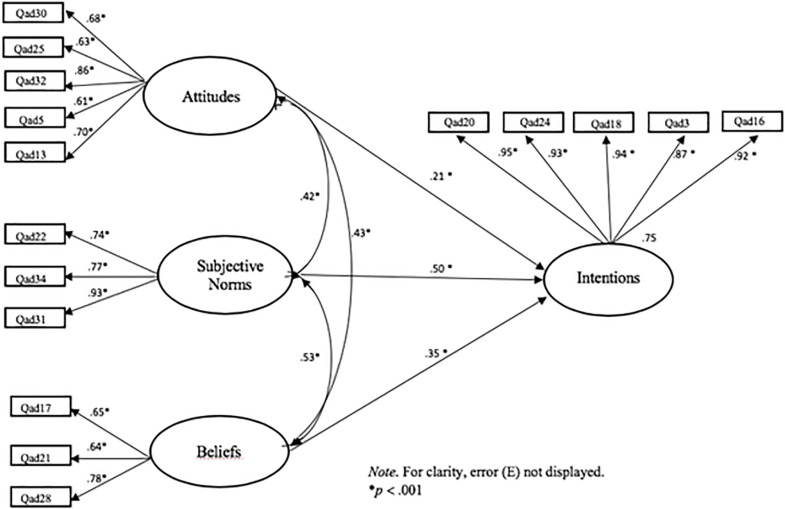
Hypothesized model on the TPB with a sample of gym users.

Results from the confirmatory analysis are presented in Figure 1. All items had statistically significant factor loadings (*p* < 0.001). The fit of the TPB model to the sample data was appropriate [χ^2^ (97) = 204.383, B-S *p* < 0.001, CFI = 0.981, TLI = 0.976, RMSEA = 0.049; with a 90% CI of 0.040 and 0.059, SRMR = 0.045]. Considering that they are part of the same framework, the TPB’s constructs were found to be correlated, with the highest correlation between subjective norms and beliefs (*r* = 0.53, *p* < 0.001) and the weakest correlation between attitudes and subjective norms (*r* = 0.42, *p* < 0.001).

Within the measurement component, all indicators were found to be reliably associated (*p* < 0.001) with their latent factors. The reliability coefficients were greater than 0.70, and all variables are close to or exceeded the level greater than 0.50 of AVE for convergent validity, ranging from 0.49 to 0.87. Indeed, the discriminant validity of all variables was supported, since the AVE estimates for each construct were larger than the interconstruct squared correlations (Fornell and Larcker, 1981). Overall, the measurement model was within the required criteria and showed good psychometric properties (Table 2).

### The Structural Model

At the structural level, results support the TPB framework in predicting intentions to PES use in our gym users sample. The overall structural model with the DWLS estimation showed a good fit to the data [χ^2^ (113) = 97.597, *p* = 0.849, CFI = 1.000, TLI = 1.00, RMSEA = 0.000, 90% CI 0.000–0.000, SRMR = 0.051]. The structural component revealed that subjective norms (β = 0.50, *p* < 0.001), beliefs (β = 0.35, *p* < 0.001), and attitudes (β = 0.21, *p* < 0.001) predicted intentions, and 75% of the variance associated with PES use intention was accounted for by its three predictors (attitudes, subjective norms, and beliefs) (Figure 1).

#### Gender

In general, the model fit for structural models was found to be satisfactory for both female [χ^2^ (98) = 207.983 (B-S *p* < 0.001), χ^2^/df = 2.122, CFI = 0.964, TLI = 0.956, RMSEA = 0.064, 90% CI 0.052–0.076, SRMR = 0.051] and male [χ^2^ (98) = 166.134 (B-S *p* < 0.001), χ^2^/df = 1.695, CFI = 0.972, TLI = 0.966, RMSEA = 0.063, 90% IC 0.046–0.079, SRMR = 0.051] subsamples. Then, we tested the hypothesis that the model was invariant across genders. As shown in Table 3, the results of the goodness-of-fit [χ^2^ (196) = 374.117 (B-S *p* < 0.001), χ^2^/df = 1.917, CFI = 0.968, TLI = 0.960, RMSEA = 0.045, 90% CI 0.038–0.052, SRMR = 0.052] for the configural invariance test indicate that the structural patterns are similar across groups. This implies that the configural model can be used as a baseline to compare with other restricted models in the invariance hierarchy. Second, metric invariance was performed by constraining the factor loadings to be equal across groups. The results of the metric invariance model [χ^2^ (208) = 398.791, (B-S *p* = 0.016), χ^2^/df = 1.917, PCFI = 0.837, CFI = 0.965, TLI = 0.960, RMSEA = 0.045, 90% CI 0.038–0.052, SRMR = 0.051] suggest a good model fit. Also, the result of the chi-square difference test between the configural model and the metric model is not significant (*p* = 0.164), and the change value of CFI (ΔCFI = 0.003) smaller than 0.01 indicates that metric invariance is achieved (Cheung and Rensvold, 2002; Byrne, 2010; Marôco, 2014). Third, a scalar invariance test was performed by restricting the intercepts across groups to be invariant. The chi-square difference between the metric model and the scalar model is not significant (*p* = 0.189) and the change value of CFI (ΔCFI = 0.005) is smaller than 0.01, suggesting that the scalar invariance hypothesis is supported (Cheung and Rensvold, 2002). Since the three invariance tests were all satisfied, the hypothesis of invariance of the predictive model across genders could not be rejected.

**TABLE 3:** Model fit indices for invariance tests in the structural model (male/*n* = 175; female/*n* = 277).

**Multigroup models**	**χ^2^**	**df**	**Δχ^2^**	**Δdf**	**B-S *p***	**CFI**	**ΔCFI**	**RMSEA**
Configural invariance	374.117	196	–	–	–	0.968	–	0.045
Metric invariance	398.791	208	24.674	12	0.016	0.965	0.003	0.045
Scalar invariance	444.072	224	45.281	16	0.001	0.960	0.005	0.047
Structural invariance	455.490	227	11.418	3	0.001	0.958	0.002	0.047

*χ^2^, chi-square; df, degrees of freedom; Δχ^2^, chi-square difference; Δdf = degrees of freedom.*

After verifying the scalar measurement invariance between genders, we tested the hypothesis that males would show more favorable attitudes, subjective norms, beliefs, and intentions to use PES than their female couterparts. *T*-tests with the Welch correction for heterokedastic variances were used to compare the mean score differences across genders. Mean score estimates are displayed in Table 4, indicating significant differences in intentions, subjective norms, and beliefs. There was no significant difference in attitudes. The mean scores of female gym users were lower than those of the male gym users by 0.61, 0.24, 0.29, and 0.26 for intention to use PES, subjective norms, beliefs, and attitudes, respectively. Effect sizes for intentions, subjective norms, beliefs, and attitudes are small (Cohen, 1988).

**TABLE 4:** Results of the gender difference analysis.

**Construct**	**M (Male; *n* = 175)**	**SD**	**M (Female; *n* = 277)**	**SD**	***t*-test (df)**	***P* value**	**Effect Size (*d*)**
Intentions	0.3694	1.923	−0.245	1.369	3.669 (284.285)	<0.001	0.367
Subjective norms	0.1447	0.853	−0.093	0.645	3.159 (297.971)	0.002	0.314
Beliefs	0.1776	0.991	−0.117	0.813	3.293 (317.383)	0.001	0.325
Attitudes	0.1595	1.498	−0.102	1.277	1.914 (327.037)	0.057	0.188

*M, Means; SD, Standard Deviation; df, degrees of freedom; *p* < 0.001 at two-tailed.*

#### Intentions and the Prediction of PES Use

**FIGURE 2:**
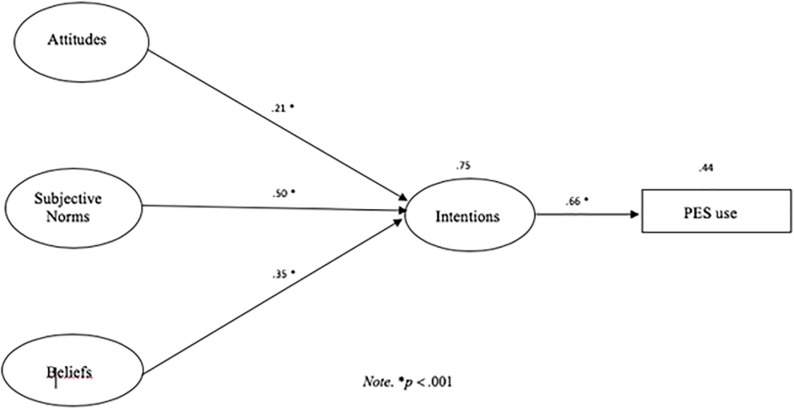
Hypothesized model on the TPB to predict PES use in gym users.

To test the hypothesis that the use of PES (self-reported) would be predicted by intentions, we used R/Lavaan for the probit regression model using the DWLS estimator appropriate for the nominal nature of the PES use variable (Figure 2). Attitudes (β = 0.21; *p* < 0.001), subjective norms (β = 0.50; *p* < 0.001), and beliefs (β = 0.35, *p* < 0.001) were signficant predictors of intentions explaining 75% of its variance (*R*^2^ = 0.752). In turn, intentions predicted the PES use (self-reported) (β = 0.66, *p* < 0.001) explaining 44% of its variance (*R*^2^ = 0.442).

## Discussion

Taking into consideration that the use of PES in fitness context is a public health concern (Tavares et al., 2019a), the current study adopts a TPB-based framework to explore the determinants of intentions to use PES in a sample of gym/fitness center users in Portugal. Because the research on PES use among gym users is limited (Molero et al., 2017), this study is partially grounded on previous research on doping in sports. Although the context and motivation related to the use of doping substances differ (e.g., performance enhancement in athletes versus improvement of bodily appearance in gym users), behaviors have similar characteristics (e.g., use of same kind of substances).

The purpose of this study was to evaluate whether the intention to use PES can be predicted by the variables considered within the TPB and whether gender influences these relationships. Based on previous findings, it was expected that TPB variables would predict intentions to use PES in our sample of gym users (e.g., Lucidi et al., 2004, 2008; Lentillon-Kaestner and Carstairs, 2010; Chan et al., 2015). As hypothezised, attitudes, subjective norms, and beliefs significantly predicted intention to use PES, accounting for almost 75% of its variance. Participants’ rejection of PES use appears to be supported by unfavorable attitudes and intentions toward the behavior and low perception of social pressure with regard to the behavior. Although in general, the beliefs were slightly negative, participants consider that consumption of these substances has some advantages. Structural component analysis revealed that subjective norms were the strongest predictor of intention to use PES, suggesting that the gym user psychosocial environment, namely, the influence of significant others, may be the primary drive of the decision to use PES. This finding is not consistent with other studies since this dimension is often the weakest predictor of intentions within the TPB (Armitage and Conner, 2001; Lucidi et al., 2004). Yet, identical results were obtained by Serpa et al. (2003) in their study with young athletes, stressing the crucial role played by social pressures and social norms, meaning that behaviors and opinion of significant others may help shape the individual’s intentions to use PES (Serpa et al., 2003; Lucidi et al., 2004). This result may be justified by possible sociocultural factors present in the current sample and by the use of a reliable multi-item scale to measure subjective norms (three items), instead of single-item measures (e.g., Lucidi et al., 2004). We also found a positive relationship between gym users’ attitudes toward PES use and intention to use PES; however, attitudes were the weakest predictor of intentions. These results, according to Jalleh et al. (2014), emphasize the complexity of the attitude-behavior relationship and the difficulty of predicting behavior. Indeed, the behavior is influenced by a variety of situational (e.g., dynamics of peer interaction, significant others and influence of role models, accessibility of PES alternatives) and environmental (e.g., easy access to PES, pharmacological and medical advancements, medicalization, political, and sociocultural context) factors that interact to promote or prevent PES use (Petróczi and Aidman, 2008).

It was also hypothesized that the predictive model would be invariant across genders (Lucidi et al., 2004). Findings from the multigroup analysis confirm this hypothesis and support the generalizability of the proposed model across gender groups. According to Lucidi et al. (2004), generalizability across groups is a powerful indicator of the model’s validity and of its capability to coherently represent the processes supporting behavioral decision making.

We also hypothesized that males were more susceptible to PES use than females. As expected, in line with other studies (Serpa et al., 2003; Pedersen, 2010; Ntoumanis et al., 2013; Backhouse et al., 2015), females believed less in the performance-enhancing effects of substances, were less susceptible to the influence of significant others, and had weaker intentions to use PES than males. This gender difference was small but statistically significant. Thus, to improve the effectiveness of PES use prevention interventions in gym users, TPB’s constructs need to be considered differently in females and males. For example, males should receive specific information about the negative health consequences of the use of these substances, including the fact that they often occur several years after their use, while females should understand the importance of maintaining a healthy and balanced eating regimen and a proper physical training program. However, in contrast with some studies (Pedersen, 2010; Ntoumanis et al., 2013; Backhouse et al., 2015), no gender differences in attitudes toward PES use were found. This apparent inconsistency with the literature may be due to contextual specificities (i.e., sports socioeconomic context), as the economic incentive is not a major motivator to initiate PES use in the fitness milieu compared to sports (Lazuras, 2016).

Finally, we hypothesized that intentions would predict self-reported use of PES. In alignment with findings of Goulet et al. (2010), our results show a significant relationship between self-reported PES use and intentions to use. In the present study, intention to use PES predicted 44% of the variance in self-reported PES use; despite the tendency for participants to underreport this behavior (Lazuras et al., 2010), the strong predictive capacity of intentions confirms the volitive self-determined characteristic of PES use (Goulet et al., 2010).

While providing insightful findings about the determinants of intentions to PES use in gym users, this study has some limitations. First, because we did not use a random-stratified sampling technique, we cannot assume that the present sample is representative of the population of Portuguese gym users, which affects generalizability of the results. Despite this, self-selected samples have been used in prior studies and may be a viable option to target specific groups (Dunn et al., 2009), such as gym users. Second, although the tested model is theoretically anchored, this study involves a cross-sectional design that precludes the inference of causality. It would be important to use experimental and longitudinal designs to make causal inferences to identify the mechanisms that contribute to the development of positive intentions to use PES and to determine how attitudes change through time. Third, PES use was assessed by self-report; hence, results may have been influenced by response bias and social desirability (Gucciardi et al., 2010). However, we attempted to avoid bias and encouraged honest responses by ensuring anonymity; specifically, participants received the questionnaires by mail and through a social network (Facebook) and completed the questionnaires in complete privacy with no personal identification being collected. Fourth, we assessed self-reported PES use only in terms of current PES use while ignoring previous use. As a consequence, we did not identify participants who had used PES before the time of the assessment or the duration of current PES use. For a more complete picture of PES use in gym users, these issues need to be considered in future studies.

Future research should also explore how different facets of the normative conduct significantly predict intentions among gym users. Identification of these facets has been considered vital to improve our understanding of the role of normative beliefs and processes not accounted by subjective norms (Lazuras et al., 2010).

The present study provides information that can contribute to building multifaceted interventions for the prevention of PES use in gym/fitness context. A major conclusion is that subjective norms are the most important predictor of intention to use PES. Taking the most important significant others into consideration (e.g., friends, training colleagues, instructors), prevention strategies may focus more efficiently on the processes of social/normative influence and on moral and ethical standards, relying on the credibility of these reference groups to promote behavior change (Donovan et al., 2002; Lucidi et al., 2004; Wiefferink et al., 2008). Additionally, interventions should provide opportunities for changes in cognitions and intentions in favor of PES use prevention (Donovan et al., 2002; Wiefferink et al., 2008; Bahrke, 2012). Strategies reinforcing the negative impact of such substances in users’ health and the promotion of the replacement of positive attitudes toward PES use into negative ones should also be implemented. Concerning preventive measures, attention should be paid to gender, personality factors (e.g., low self-confidence, perfectionism), and PES users/non-users’ expectancies (e.g., positive expectancies regarding the use of these substances and identification with peers who promote health risk behavior) as influencing factors of PES use susceptibility. Interventions targeting persistent users should also ensure social support and the opportunity to reduce health risks (e.g., using health checkups, develop awareness about the severity of associated ill-health conditions).

## Data Availability Statement

The datasets for this article are not publicly available because the datasets generated during the current study are available from the corresponding author on reasonable request. Requests to access the datasets should be directed to ana.tavares@estesl.ipl.pt.

## Ethics Statement

This study was carried out in accordance with the recommendations of the Ethical Committee of the Faculty of Human Kinetics. All subjects gave written informed consent in accordance with the Declaration of Helsinki. The protocol was approved by the Ethical Committee of the Faculty of Human Kinetics.

## Author Contributions

AT and SS were enrolled in the study design and data collection. AT, SS, and LC wrote the first draft of the manuscript. AT, AR, and JM participated in the data analysis and wrote the methodology and results. AT, SS, AR, JM, and LC participated in the final revisions of the manuscript, read and approved the final version of the manuscript, and agreed with the order of the presentation of the authors.

## Conflict of Interest

The authors declare that the research was conducted in the absence of any commercial or financial relationships that could be construed as a potential conflict of interest.
